# Onset of analgesia with ibuprofen sodium in tension-type headache: a randomized trial

**DOI:** 10.1186/s40780-015-0012-9

**Published:** 2015-04-02

**Authors:** Elias Packman, Rina Leyva, David Kellstein

**Affiliations:** Institute for Applied Pharmaceutical Research, 214 Sycamore Avenue, Merion Station, PA 19066 USA; Pfizer Consumer Healthcare, 1 Giralda Farms, Madison, NJ 07940 USA

**Keywords:** Ibuprofen sodium, Fast-absorbed ibuprofen, Tension headache, Analgesia, Over the counter

## Abstract

**Background:**

Ibuprofen is known to be efficacious in the treatment of tension-type headache, the most common form of primary headache. A novel tablet formulation of ibuprofen sodium is more rapidly absorbed than standard ibuprofen. This study evaluated onset of analgesia and overall efficacy of ibuprofen sodium in episodic-type tension headache (ETTH) compared with standard ibuprofen and placebo.

**Methods:**

This randomized, double-blind, single-center, parallel-group study included adults aged 18–65 years with ≥4 moderately severe ETTHs per month for 6 months. Within 45 minutes of onset of at least moderately severe ETTH, subjects were randomized 2:2:1 to receive a single oral dose of ibuprofen sodium tablets (Advil® Film Coated; 2 × 256 mg [equivalent to 400 mg standard ibuprofen]), standard ibuprofen tablets (Motrin®; 2 × 200 mg), or placebo. The coprimary end points were time-weighted sum of pain relief rating and pain intensity difference scores over 3 hours (SPRID 0–3) and time to meaningful pain relief (MPR) assessed by double-stopwatch method.

**Results:**

A total of 226 subjects were randomized to ibuprofen sodium (n = 91), standard ibuprofen (n = 89), and placebo (n = 46). Demographics and baseline characteristics were comparable between treatment groups. Mean SPRID 0–3 scores were significantly superior (*P* < .001) for ibuprofen sodium (9.6) and standard ibuprofen (9.8) versus placebo (3.5), but were not significantly different from each other (*P* = .812). Time to MPR was significantly (*P* < .001) shorter for ibuprofen sodium and standard ibuprofen compared with placebo (median 40.6, 48.5, and >180 minutes, respectively). Time to MPR was numerically faster for ibuprofen sodium than standard ibuprofen. This difference was not statistically significant (*P* = .253) using the protocol-specified analysis but was (*P* = .022) in a post hoc analysis using the Gehan-Wilcoxon test, which assigns higher weights to earlier events. (The post hoc analysis was performed because Kaplan-Meier graphs and results for time to first perceptible relief favored ibuprofen sodium over standard ibuprofen at earlier time points.) There were no adverse events.

**Conclusions:**

This novel ibuprofen sodium tablet provided rapid, efficacious relief of ETTH and was well tolerated.

**Trial registration:**

ClinicalTrials.gov NCT01362491.

## Background

Tension-type headache is the most common type of primary headache, with a lifetime prevalence in the general population ranging from 30% to 78% [1]. Diagnostic criteria for episodic tension-type headache (ETTH), as per *The International Classification of Headache Disorders*, 2nd edition, include having at least 10 headache episodes (lasting 30 minutes to 7 days) occurring on fewer than 15 days per month over a period of at least 3 months [1]. Additionally, at least 2 of the following items must be present for a diagnosis of ETTH: bilateral location, a feeling of pressure or tightening, mild or moderate intensity, and lack of exacerbation by routine physical activity [1].

Ibuprofen (IBU) is a nonsteroidal anti-inflammatory drug (NSAID) that is widely available without a prescription (i.e., over the counter [OTC]) for the relief of acute pain resulting from various causes, including headache [2]. In many countries, nonprescription IBU labeling dictates using a maximum single dose of 400 mg, which can be administered up to 3 times per day as needed. Several well designed clinical studies have demonstrated the efficacy of OTC IBU in the treatment of tension-type headache [3], dental pain [4,5], sore throat [6], and postpartum episiotomy pain [7].

When acute pain necessitates a pharmacologic intervention, pain sufferers desire relief as quickly as possible. Serum concentrations of IBU are highly correlated with the level of analgesia [8]. Pharmacologic enhancements increasing IBU solubility in water facilitate gastric and enteric mucosal absorption, potentially leading to a faster onset of pain relief [9]. Considerable efforts have been expended to develop faster-absorbed formulations of IBU for that purpose. These efforts include the development of soft gelatin capsules containing solubilized IBU [10] and salt forms of IBU, e.g., arginine [9,11], lysine [10], and sodium [12] salts. Several studies have confirmed that IBU salt formulations provide a clinical advantage of more rapid pain relief in comparison to standard IBU in subjects with postsurgical dental pain [9,11,13-15].

Most recently, a novel immediate-release tablet containing 256 mg of IBU sodium dihydrate (IBU_Na_; equivalent to 200 mg of IBU free acid) has been developed. Pharmacokinetic studies have demonstrated that this formulation of IBU_Na_ is absorbed faster than standard IBU tablets, and as fast as both solubilized IBU and IBU lysine [16]. In a clinical study of postsurgical dental pain, the pharmacokinetic advantage of IBU_Na_ tablets was translated into more rapid pain relief than that achieved with standard IBU tablets [17]. In order to gain further insight into the efficacy of IBU_Na_ in an additional pain state, the current study (NCT01362491) evaluated the onset, and overall analgesic efficacy, of a single oral dose of IBU_Na_ tablets in comparison with standard IBU and placebo in the treatment of ETTH when both active treatments were administered at a dose that was equivalent to 400 mg of IBU acid. Our hypothesis was that IBU_Na_ would provide a faster onset of analgesia compared with standard IBU tablets and have superior overall analgesic efficacy compared with placebo.

## Methods

### Subjects and study design

This single-center, randomized, double-blind (third-party blind), 3-hour, single-dose, placebo-controlled, parallel-group study was conducted from June 21, 2011, through March 9, 2012. Healthy men and women (aged 18 to 65 years) who had a diagnosis of ETTH as defined by the International Headache Society [1] were enrolled. The headaches had to have begun prior to age 50, be of at least moderate severity, have a frequency of ≥4 headache episodes per month for 6 months, typically respond to OTC analgesics, and to last for ≥3 hours if left untreated. Women could not be pregnant or lactating and a medically approved method of contraception was required for women who were premenopausal or had been postmenopausal for less than 2 years. Subjects were excluded if the Investigator determined that the presence or history of a significant medical condition increased the risk for that subject; if there was a presence or history of alcohol abuse (≥3 drinks per day on a regular basis), gastrointestinal bleeding or ulcer, bleeding disorder, or substance abuse within the past 2 years; and/or if there was treatment for depression within the past 6 months. Having a history of recurrent migraine headache (defined as, on average, ≥1 migraine headache per month over the past 6 months), menstrual headaches, or habituation to analgesic medications (i.e., routine use of oral analgesics ≥5 times per week) were also exclusionary. Potential subjects were ineligible if they had taken any type of analgesic, antipyretic, sedative, or vasoactive agent within 12 hours of study drug administration, or if they had ingested any caffeine-containing beverages, chocolate, or alcohol within 4 hours of study drug. Subjects with a hypersensitivity to any OTC NSAID or acetaminophen or who were taking concomitant medication contraindicated with an NSAID were ineligible.

Subjects meeting the eligibility requirements were instructed to present to a single clinical research center (Institute for Applied Pharmaceutical Research, Philadelphia, PA), within 45 minutes of onset of an ETTH. Presenting subjects were screened for baseline pain severity using a 4-Point Categorical Pain Severity Rating (PSR) Scale (0 = none, 1 = mild, 2 = moderately severe, 3 = severe) and a 100-mm visual analog pain scale (VAS). Subjects with at least moderately severe baseline headache pain, defined as a score ≥2 on the categorical PSR and confirmed by score ≥66 mm on the VAS, were eligible for randomization. All subjects provided written informed consent prior to screening and enrollment. The study was approved by the Sterling Institutional Review Board (Atlanta, GA) and was conducted in accordance with International Conference on Harmonization Good Clinical Practice Guidelines and the guiding principles of the Declaration of Helsinki and amendments thereof.

### Treatment interventions and administration

Subjects were stratified by baseline pain (moderately severe or severe) and gender and randomized in a ratio of 2:2:1 according to a computer-generated randomization schedule (supplied by Pfizer Consumer Healthcare Biostatistics Department, Madison, NJ) to receive a single oral dose of IBU sodium dihydrate (new Advil^®^ Film-Coated Tablets [IBU_Na_]; 2 × 256 mg; equivalent to 400 mg standard IBU; Pfizer Consumer Healthcare), standard IBU tablets (Motrin^®^ IB [IBU_Mot_]; 2 × 200 mg; McNeil Consumer Healthcare, Fort Washington, PA), or placebo (2 tablets). Study drug was administered with 8 ounces of water. Since the study medications did not have an identical appearance, subjects were blindfolded during treatment administration. Aside from a disinterested third party who retained sole possession of the randomization codes and administered the treatments, all study personnel and subjects were blinded to the identity of the study drug. Efficacy was evaluated for 3 hours postdose. Rescue medication, at the Investigator’s discretion, was permitted for subjects not experiencing adequate pain relief within 2 hours of study treatment administration; any subjects requiring rescue medication prior to 2 hours were discontinued from the study.

### Efficacy and safety assessments

The double-stopwatch method was used to evaluate time to first perceptible pain relief (FPR) and time to meaningful pain relief (MPR). The double-stopwatch method is the FDA-preferred method for assessing onset of analgesia [18] and has been employed extensively in trials of headache, dental pain, and other pain models [17,19-23]. At the time of treatment administration, the study coordinator or designee started 2 blinded stopwatches. The subject was given the first stopwatch and told to stop it as soon as any headache relief was experienced. Once the subject stopped the first stopwatch, the elapsed time was recorded as time to FPR and the subject was asked whether their relief was “meaningful.” If the subject answered affirmatively, then the time to FPR was also considered the time to MPR. However, if the subject did not consider the FPR to be meaningful, he/she was given the second stopwatch and asked to stop it when “meaningful relief” was achieved; in this scenario, elapsed time on the second stopwatch was recorded as time to MPR.

At 1, 2, and 3 hours postdose or at the time of rescue medication use (if applicable), subjects were asked to rate their pain severity on the 4-point PSR and their pain relief on a 5-point Categorical Pain Relief Rating (PRR) Scale (0 = no relief, 1 = a little relief, 2 = some relief, 3 = a lot of relief, 4 = complete relief).

Safety was monitored throughout the study. All adverse events (AEs), observed or volunteered, were to be recorded using Medical Dictionary for Regulatory Activities (MedDRA) version 13.0, and severity was noted. Vital signs (i.e., blood pressure, heart rate, and respiratory rate) were measured at baseline and again at study end or at the time of rescue medication administration (if applicable). No laboratory evaluations were performed.

### Study end points

There were 2 coprimary end points: (1) mean time-weighted sum of PRR and Pain Intensity Difference (PID) scores from 0 through 3 hours (SPRID 0–3) for IBU_Na_ versus placebo and (2) time to MPR for IBU_Na_ versus IBU_Mot_. The primary hypothesis in this superiority study was that a single oral dose of IBU_Na_ would be more efficacious than placebo in the treatment of ETTH. The coprimary hypothesis was that IBU_Na_ would have a faster onset of action, as assessed by time to MPR, than IBU_Mot_ in the treatment of ETTH.

Secondary efficacy end points included time to MPR for the remaining comparisons and time to FPR (confirmed by MPR) for all pairwise comparisons. Other secondary assessments (all pairwise comparisons) included PRR, PID, and sum of PRR and PID (PRID) scores at 1, 2, and 3 hours postdose. The following summary efficacy scores were also compared over 2 and 3 hours postdose for all nonprimary pairwise comparisons: sum of PID scores (SPID 0–2 and 0–3), time-weighted sum of PRR scores (TOTPAR 0–2 and 0–3), and SPRID 0–2 and 0–3. The cumulative proportions of subjects achieving MPR and FPR were assessed at 0.5, 1, 2, and 3 hours postdose, and the cumulative proportions achieving complete relief were assessed at 1, 2, and 3 hours. Additional secondary assessments included duration of pain relief as measured by time to treatment failure (i.e., time to rescue medication use or discontinuation due to lack of efficacy) and cumulative proportion of treatment failures at 1, 2, and 3 hours. Safety end points included frequency and severity of treatment-emergent AEs and frequency of serious AEs.

### Power calculation and sample size

An estimated sample size of 225 subjects (90 subjects in each active treatment group and 45 in the placebo group) was needed to provide at least 80% power at the 5% level of significance (2-sided) to allow detection of a 2.3-unit difference in SPRID 0–3 between IBU_Na_ and placebo and to detect a hazard ratio (HR) of 1.6 for comparison of time to MPR between IBU_Na_ and IBU_Mot_.

### Statistical analyses

Efficacy analyses were based on the intent-to-treat (ITT) population, which included all randomized subjects who received study drug and provided a baseline assessment. The safety population consisted of all subjects who took study medication.

PID, SPID, PRID, SPRID, PRR, and TOTPAR were analyzed using analysis of variance (ANOVA), with treatment, gender, and baseline pain severity terms included in the model. Least-squares means from the ANOVA model were used to compute 95% confidence intervals (CIs) for each pairwise treatment difference. The distribution of time to MPR, time to FPR (confirmed by time to MPR to minimize placebo response), and time to treatment failure were analyzed using the proportional hazards regression model, with terms for treatment, gender, and baseline pain severity in the model. The Cochran-Mantel-Haenszel (CMH) test, controlling for gender and baseline pain severity, was used to analyze the cumulative proportions of subjects with MPR, FPR, complete relief, and treatment failure at each specified time point. In addition to the prespecified statistical analysis, a post hoc analysis evaluated time to event data (time to FPR and MPR) using the Gehan-Wilcoxon test, which assigns higher weights to earlier events. This is in contrast to the proportional hazards regression of the CMH test, which assigns equal weight to all events.

All statistical computations were performed using SAS version 9.2 (SAS Institute, Cary, NC). Two-sided between-group tests were conducted at the 5% level of significance. For protection from type I error due to multiple end points, the primary end points were tested sequentially. For protection from type I error due to multiple comparisons, each pairwise comparison was eligible for significance only if the corresponding overall treatment effect among the 3 groups was also significant.

The authors had full access to all the data in the study and take responsibility for the integrity of the data and the accuracy of the data analysis.

## Results

### Subject disposition and baseline characteristics

**Figure 1:**
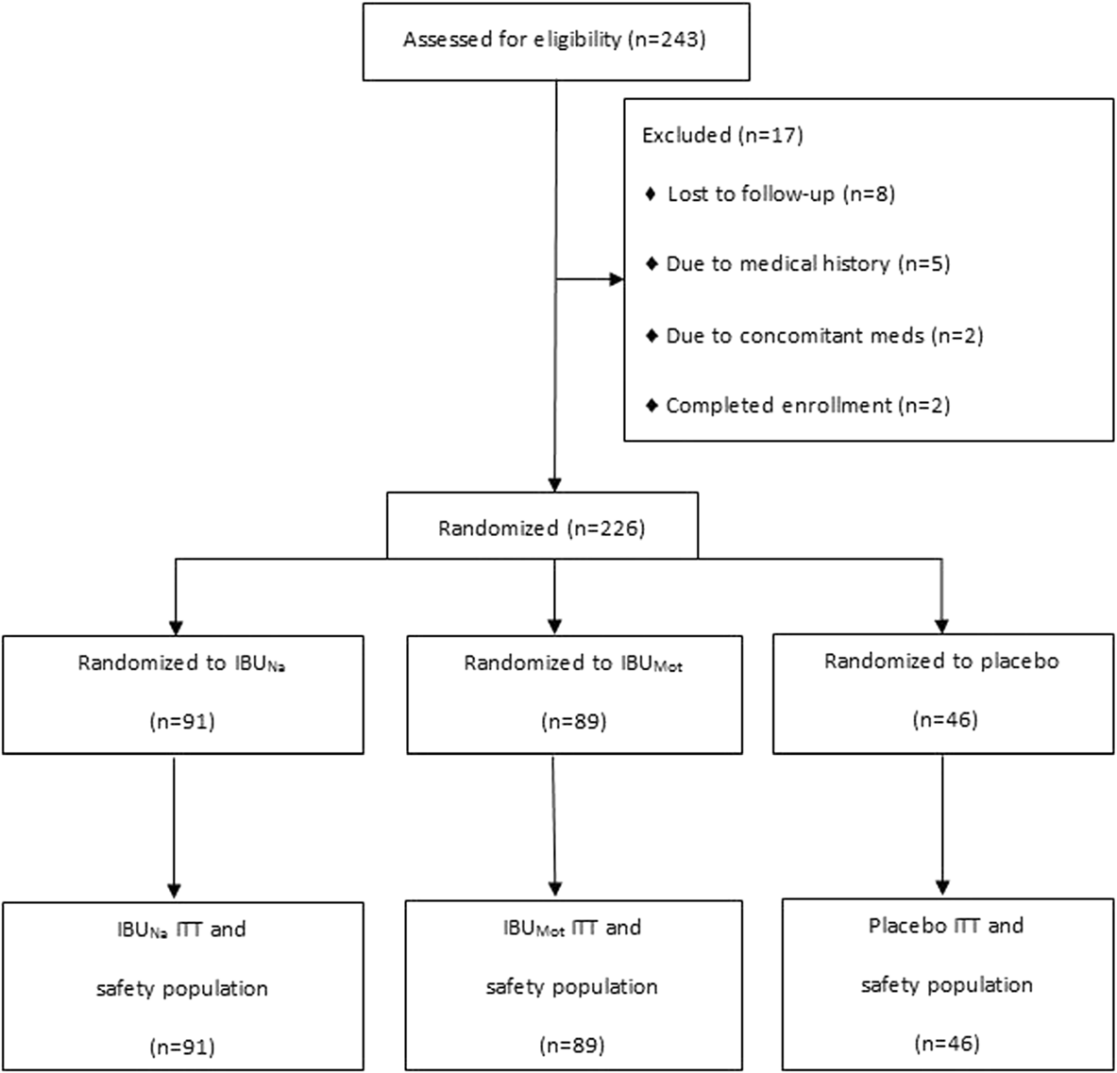
**Subject disposition.** IBU_Mot_, Motrin^®^; IBU_Na_, ibuprofen sodium dihydrate; ITT, intent-to-treat.

Of 243 screened subjects, 226 were randomized to IBU_Na_ (n = 91), IBU_Mot_ (n = 89), and placebo (n = 46). All of the randomized participants received study medications and completed the study (i.e., there were no early withdrawals); thus, all 226 randomized participants constituted the ITT and safety populations (Figure 1). Most subjects were women, nearly all were Caucasian, and the average subject age was 42.8 years (range: 18–65). Treatment groups were comparable for all demographic characteristics (Table 1).

**Table 1:** **Baseline and demographic characteristics: randomized subjects**

	**Placebo (n = 46)**	**IBU**_**Na**_ **(n = 91)**	**IBU**_**Mot**_ **(n = 89)**
Gender, n (%)			
Male	16 (34.8)	31 (34.1)	30 (33.7)
Female	30 (65.2)	60 (65.9)	59 (66.3)
Race, n (%)			
White/Caucasian	46 (100.0)	88 (96.7)	86 (96.6)
Black	0	3 (3.3)	3 (3.4)
Ethnicity, n (%)			
Non-Hispanic	46 (100.0)	91 (100.0)	89 (100.0)
Age, mean (SD), y	39.9 (15.2)	42.3 (14.3)	44.8 (13.5)
Time with recurrent headaches, mean (SD), y	12.3 (9.0)	12.8 (8.7)	13.2 (7.6)
Tension headache frequency, past 6 months, mean (SD)	5.5 (1.7)	5.9 (2.3)	6.0 (2.2)
Pain VAS, mean (SD), mm	82.1 (9.0)	82.1 (8.9)	81.9 (8.4)
Categorical pain severity, n (%)			
Moderately severe	36 (78.3)	73 (80.2)	70 (78.7)
Severe	10 (21.7)	18 (19.8)	19 (21.3)

IBU_Mot_, Motrin^®^; IBU_Na_, ibuprofen sodium; SD, standard deviation; VAS, visual analog scale.

### Efficacy

#### Primary efficacy

##### SPRID 0–3

**Figure 2:**
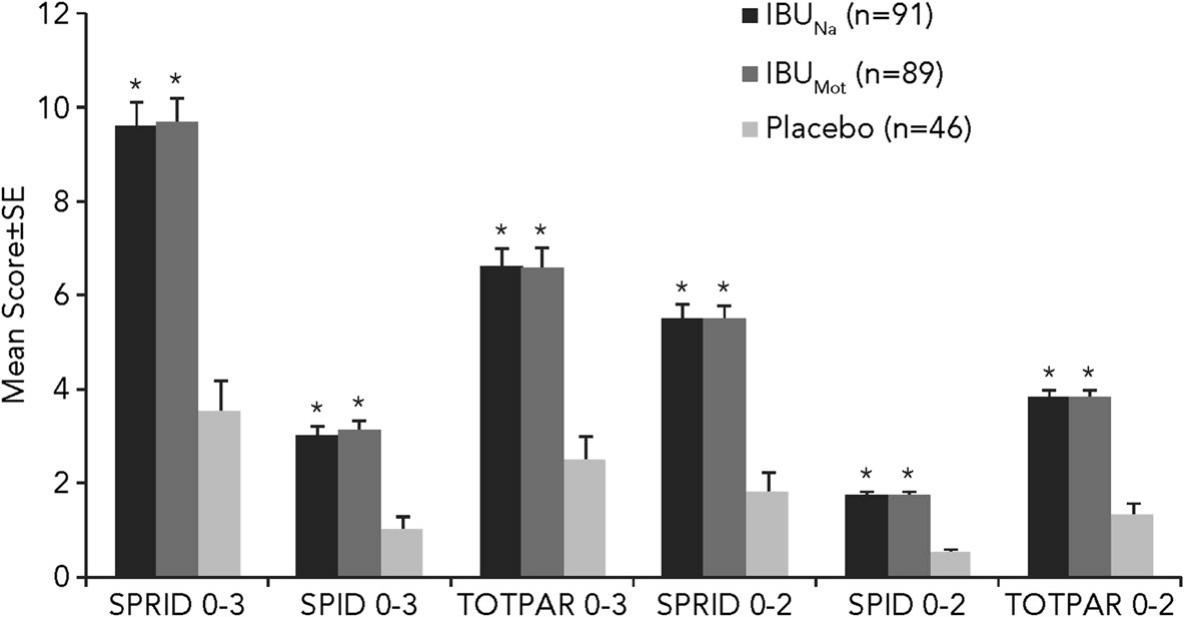
**Two- and 3-hour summary efficacy measures.** *P ≤ .001 vs placebo; IBU_Mot_, Motrin^®^; IBU_Na_, ibuprofen sodium dihydrate; SE, standard error; SPID, sum of pain intensity difference scores; SPRID, time-weighted sum of Pain Relief Rating and pain intensity difference scores; TOTPAR, time-weighted sum of Pain Relief Rating scores; 0–3, from time 0 to 3 hours after study administration; 0–2, from time 0 to 2 hours after study administration.

The mean SPRID 0–3 scores were 9.6, 9.8, and 3.5 for IBU_Na_, IBU_Mot_, and placebo, respectively (Figure 2). The SPRID 0–3 score was statistically significantly better for IBU_Na_ versus placebo (treatment difference 6.11; 95% CI 4.49–7.73; *P* < .001). Likewise, IBU_Mot_ also had a SPRID 0–3 score that was statistically significantly better than placebo (treatment difference 6.27; 95% CI 4.65–7.89; *P* < .001). The SPRID 0–3 scores for IBU_Na_ and IBU_Mot_ were not significantly different from each other (treatment difference −0.16; 95% CI −1.49–1.17; *P* = .812). Thus, as expected, both active treatments provided superior pain relief compared with placebo.

##### Time to MPR

**Figure 3:**
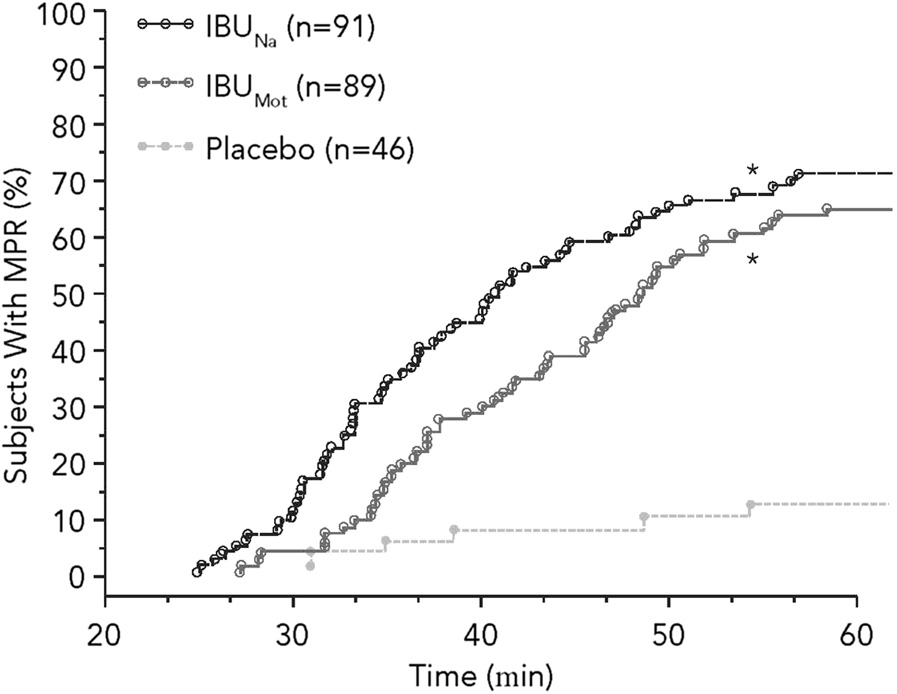
**Time to meaningful pain relief (hour 0–1).** *P < .001 vs placebo; IBU_Mot_, Motrin^®^; IBU_Na_, ibuprofen sodium dihydrate; MPR, meaningful pain relief.

Median time to MPR was significantly faster for IBU_Na_ (40.6 minutes; HR 4.38; 95% CI 2.69–7.14; *P* < .001) and for IBU_Mot_ (48.5 minutes; HR 3.64; 95% CI 2.23–5.94; *P* < .001) when compared to placebo (>180 minutes). The shorter time to MPR noted for IBU_Na_ compared with IBU_Mot_ was not statistically significant using the prespecified analysis (HR 1.20; 95% CI 0.88–1.65; *P* = .253; Figure 3). However, using a post hoc analysis (Gehan-Wilcoxon test), which assigns higher weights to earlier events, MPR was reached significantly faster with IBU_Na_ compared with IBU_Mot_ (*P* = .022).

#### Secondary efficacy

##### Time to FPR

**Figure 4:**
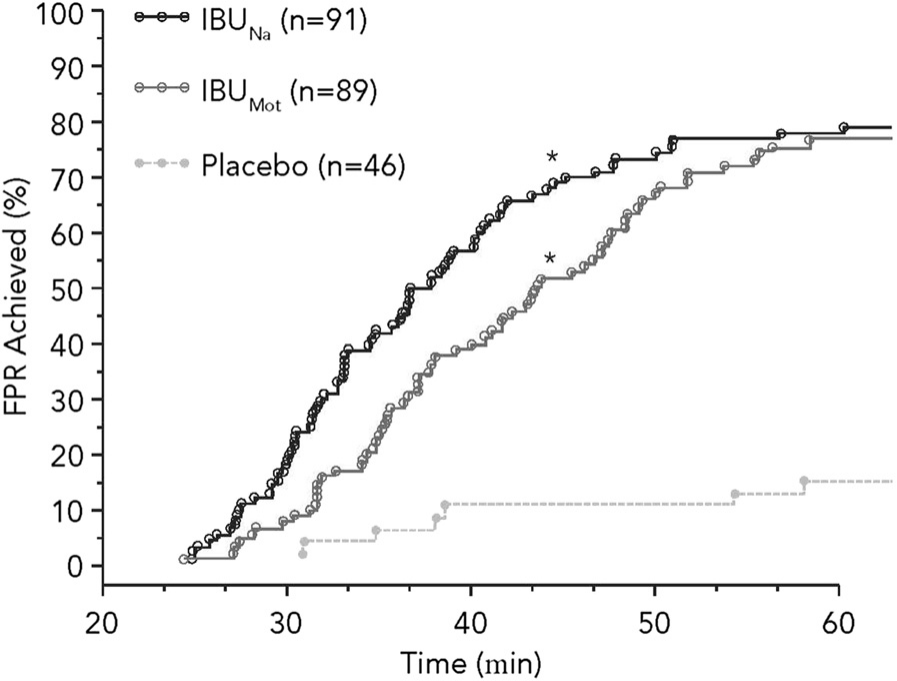
**Time to first perceptible pain relief (hour 0–1).** *P < .001 vs placebo; FPR, first perceptible relief; IBU_Mot_, Motrin^®^; IBU_Na_, ibuprofen sodium dihydrate.

Median time to FPR was 36.9 minutes for IBU_Na_, 43.6 minutes for IBU_Mot_, and >180 minutes for placebo (Figure 4). Compared with placebo, both IBU_Na_ (HR 4.68; 95% CI 2.87–7.64; *P* < .001) and IBU_Mot_ (HR 3.88; 95% CI 2.38–6.34; *P* < .001) had significantly faster times to FPR. Time to FPR was not significantly different between IBU_Na_ and IBU_Mot_ using the prespecified analysis (HR 1.21; 95% CI 0.88–1.66; *P* = .247); however, in the post hoc analysis (Gehan-Wilcoxon test), IBU_Na_ provided a significantly faster time to FPR compared with IBU_Mot_ (*P* = .018).

### PRID

**Figure 5:**
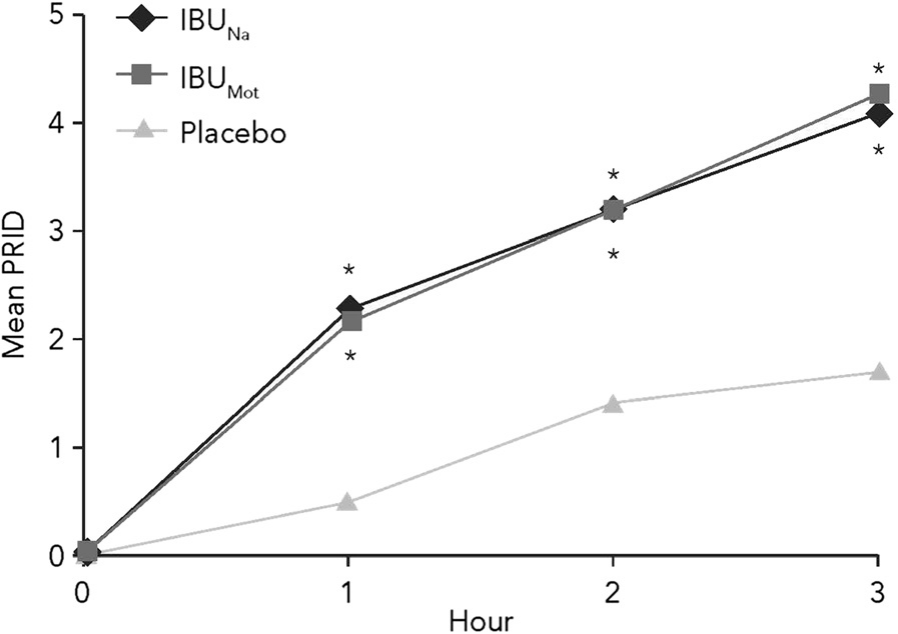
**Sum of pain relief rating and pain intensity difference (PRID) scores over time.** *IBU_Na_ and IBU_Mot_ both *P* < .001 vs placebo; IBU_Mot_, Motrin^®^; IBU_Na_, ibuprofen sodium dihydrate.

The PRID (sum of PRR and PID) scores were significantly better for both IBU_Na_ and IBU_Mot_ versus placebo at 1, 2, and 3 hours (Figure 5; *P* < .001 for all comparisons), providing further evidence that both active treatments provided significant pain relief and reductions in pain intensity. IBU_Na_ and IBU_Mot_ were not significantly different from each other with regard to PRR, PID, and PRID scores at any time point.

### SPRID 0–2 and other summary efficacy scores

Results for the other summary efficacy end points, consisting of 0–2- and 0–3-hour SPID, TOTPAR, and SPRID, were consistent with those for SPRID 0–3 (i.e., IBU_Na_ and IBU_Mot_ were significantly better than placebo [*P* < .001] for each measure but not significantly different from each other) (Figure 2). Results of primary and secondary efficacy end points stratified by baseline pain status (moderately severe and severe) were generally consistent with those observed in the overall ITT population; therefore, these subgroup analyses are not shown.

### Cumulative proportions of subjects attaining relief

Cumulative proportions of subjects achieving FPR or MPR by 0.5, 1, 2, and 3 hours are shown in Table 2. At 30 minutes postdose, 18.7% of the IBU_Na_-treated group, 7.9% of the IBU_Mot_-treated group, and 0 subjects in the placebo group reported FPR. At this time point, significantly more subjects treated with IBU_Na_ experienced FPR compared with either placebo (treatment difference 18.58, 95% CI 10.43–26.73; *P* = .002) or IBU_Mot_ (treatment difference 10.91; 95% CI 0.98–20.83; *P* = .033). The treatment difference for FPR at 30 minutes between IBU_Mot_ and placebo (treatment difference 7.80; 95% CI 2.14–13.46; *P* = .055) trended towards statistical significance. Meaningful pain relief was reported by 12.1% of the IBU_Na_ group, 4.5% of the IBU_Mot_ group, and 0 subjects in the placebo group by 30 minutes postdose. This difference in MPR was significant for IBU_Na_ versus placebo (treatment difference 12.02; 95% CI 5.27–18.77; *P* = .015) but not for IBU_Mot_ vs placebo (treatment difference 4.50; 95% CI 0.13–8.88; *P* = .146); the difference in MPR for IBU_Na_ versus IBU_Mot_ at 30 minutes was borderline significant (treatment difference 7.62; 95% CI: −0.38–15.63; *P* = .064).

**Table 2:** **Cumulative proportion of subjects achieving relief at 0.5, 1, 2, and 3 hours**

	**Proportion of subjects achieving end point, %**			**Treatment difference (95% CI)** ***P*** **value** ^**a**^		
	**IBU**_**Na**_	**IBU**_**Mot**_	**Placebo**	**IBU**_**Na**_ **vs Placebo**	**IBU**_**Na**_ **vs IBU**_**Mot**_	**IBU**_**Mot**_ **vs Placebo**
**30 minutes**						
FPR	18.7	7.9	0	**18.58 (10.43–26.73)** ***P*** **= .002**	**10.91 (0.98–20.83)** ***P*** **= .033**	7.80 (2.14–13.46) *P* = .055
MPR	12.1	4.5	0	**12.02 (5.27–18.77)** ***P*** **= .015**	7.62 (−0.38–15.63) *P* = .064	4.50 (0.13–8.88) *P* = .146
**1 hour**						
FPR	76.9	74.2	15.2	**62.03 (48.65–75.41)** ***P*** **< .001**	2.96 (−9.52–15.44) *P* = .642	**59.44 (45.69–73.19)** ***P*** **< .001**
MPR	71.4	65.2	13.0	**58.58 (44.96–72.21)** ***P*** **< .001**	6.40 (−7.27–20.07) *P* = .357	**52.31 (38.20–66.41)** ***P*** **< .001**
Complete relief	0	0	0	NA	NA	NA
**2 hours**						
FPR	85.7	86.5	45.7	**40.26 (23.85–56.68)** ***P*** **< .001**	−0.72 (−10.89–9.45) *P* = .889	**41.19 (24.78–57.59)** ***P*** **< .001**
MPR	85.7	86.5	39.1	**46.68 (30.43–62.93)** ***P*** **< .001**	−0.72 (−10.89–9.45) *P* = .889	**47.59 (31.35–63.83)** ***P*** **< .001**
Complete relief	5.5	4.5	2.2	3.21 (−3.12–9.55) *P* = .389	0.98 (−5.45–7.41) *P* = .764	2.35 (−3.69–8.39) *P* = .490
**3 hours**						
FPR	85.7	86.5	45.7	**40.26 (23.85–56.68)** ***P*** **< .001**	−0.72 (−10.89–9.45) *P* = .889	**41.19 (24.78–57.59)** ***P*** **< .001**
MPR	85.7	86.5	45.7	**40.26 (23.85–56.68)** ***P*** **< .001**	−0.72 (−10.89–9.45) *P* = .889	**41.19 (24.78–57.59)** ***P*** **< .001**
Complete relief	37.4	38.2	8.7	**28.71 (15.72–41.70)** ***P*** **< .001**	−0.93 (−15.22–13.36) *P* = .898	**29.37 (16.19–42.56)** ***P*** **< .001**

^a^Treatment difference (first treatment – second treatment) and its associated CI were calculated based on the Cochran-Mantel-Haenszel (CMH) adjusted proportions and the corresponding standard errors; *P* values from the CMH test, controlling for baseline Pain Severity Rating and gender. CI, confidence interval; FPR, first perceptible relief; IBU_Mot_, Motrin^®^; IBU_Na_, ibuprofen sodium; MPR, meaningful pain relief; NA, not applicable. Statistically significant differences are indicated by bold type.

By 1 hour, proportions of subjects attaining FPR and MPR were significantly greater for both active treatment groups versus placebo (*P* < .001). At hour 3 (end of study), significantly more subjects in both active treatment groups had achieved FPR, MPR, and complete relief versus placebo (*P* < .001 for all comparisons).

### Treatment failure

No subject took rescue medication or discontinued due to lack of efficacy. Therefore, time to treatment failure could not be assessed.

### Safety evaluation

Both formulations of IBU were well tolerated. There were no treatment-emergent AEs, deaths, serious AEs, or significant changes in vital signs reported during the study.

## Discussion

The novel IBU formulation described herein was developed as an immediate-release tablet formulation containing 256 mg of IBU_Na_ as the dihydrate salt. This formulation provides 200 mg of IBU free acid, which is the amount in currently marketed OTC IBU tablets in the United States and many other countries. In this study, as expected, both IBU_Na_ and IBU_Mot_ provided significantly better relief of ETTH than placebo across multiple efficacy end points and provided equivalent overall pain relief. No AEs were reported in the study, and all treatments were well tolerated. The methodology used in this ETTH study mirrors that of a previously published study, which showed that solubilized IBU provided significantly faster, and superior, pain relief than acetaminophen tablets (*P* ≤ .02) [19].

According to the primary protocol-specified analysis, IBU_Na_ did not provide statistically significantly faster relief of ETTH than IBU_Mot_. However, IBU_Na_ was numerically faster than IBU_Mot_ by 6.7 minutes for onset of FPR and by 7.9 minutes for onset of MPR. In a post hoc analysis using the Gehan-Wilcoxon test, which assigns higher weights to earlier events, these differences were statistically significant. The post hoc analysis was performed because results of earlier studies using the third molar extraction model of dental pain showed that IBU_Na_ provides a more rapid onset of analgesic effect compared with other standard IBU products [14,15,17] and because, in the current study, the Kaplan-Meier graphs and results for percentage of subjects reporting FPR confirmed by MPR showed trends and significant differences, respectively, favoring IBU_Na_ over IBU_Mot_ at earlier time points. Results from the analyses of cumulative proportions of subjects achieving relief also suggest an advantage for IBU_Na_ during the first 30 minutes postdose: more subjects in the IBU_Na_ group attained confirmed FPR within 30 minutes compared with IBU_Mot_, and the proportion with MPR was significantly different from placebo starting at 30 minutes for IBU_Na_ whereas the difference versus placebo did not become significant until 1 hour postdose for IBU_Mot_.

Prior studies evaluating other formulations of IBU_Na_ in postoperative dental pain models have demonstrated that IBU_Na_ has a faster onset of analgesia relative to standard IBU. Schleier et al. [14] randomized 396 subjects who had undergone third molar extraction to IBU_Na_ (2 × 256 mg, equivalent to 400 mg standard IBU) or standard IBU (2 × 200 mg). Onset of pain relief occurred earlier and relief increased faster in the IBU_Na_ group, and the median time to substantial pain relief was 42 minutes with IBU_Na_ versus 56 minutes with standard IBU. Similarly, Norholt et al. [15] randomized 144 subjects to receive a single dose of either IBU_Na_ or standard IBU following third molar extraction on the first surgical day and then were crossed over to the opposite treatment following removal of a second third molar on the second surgical day. In this study, IBU_Na_ produced onset of pain relief 6 minutes faster (*P* = .004) and provided substantial relief within 30 minutes for twice as many subjects (11% versus 5%) as did standard IBU.

The formulation of IBU_Na_ used in the current study was also previously assessed in the third molar extraction model of dental pain. Brain et al. [17] randomized 316 subjects to receive IBU_Na_ (2 × 256 mg), 2 different formulations of standard IBU (2 × 200 mg), or placebo, and monitored subjects for 8 hours postdose. Time to MPR, 1 of 2 coprimary end points, was significantly (*P* < .001) faster with IBU_Na_ (median 42.4 minutes) than with placebo (>8 hours), pooled standard IBU (55.3 minutes) or IBU_Mot_ (60.7 minutes). As expected, IBU_Na_ was associated with a SPRID 0–8 score that was significantly better than placebo *(P* < .001) and comparable to standard IBU [17]. The discrepancy between results seen in the dental pain model and the current study in ETTH could be due to a difference in model sensitivity. The third molar extraction model of dental pain is the most widely used model for assessing acute analgesia and has well established model sensitivity and reproducibility [24]. The surgical methodology is standardized and the resultant levels of postsurgical pain are probably much higher and less variable than those in tension headache, which is a naturalistic as opposed to an induced, postsurgical pain model.

The current study extends the findings of rapid onset of analgesic efficacy with IBU_Na_ observed in dental pain studies to ETTH. Rapid onset of analgesia is a highly desirable attribute in the treatment of ETTH, the most common type of primary headache. ETTH affects up to three-quarters of the population at some point in their lives [1], causing suffering and contributing to office visits, hospital admissions, and absenteeism [25]. Safe, effective, and fast-acting treatments may help reduce ETTH-related disability and improve quality of life.

There are potential limitations of this study. The study population was very homogeneous, with 97% white/Caucasian subjects, which may limit extrapolation of results to the general population. However, the authors are not aware of any racial differences in the response to IBU or other NSAIDs for the treatment of tension headache. Another possible limitation is the external validity in relation to other types of pain. However, OTC IBU and other NSAIDs are efficacious in both the treatment of tension headache and other types of acute pain, and this particular formulation of IBU with sodium has also been demonstrated to be efficacious and fast-acting in the treatment of postsurgical dental pain [17].

## Conclusions

In conclusion, this single-dose study indicates that a novel IBU_Na_ tablet formulation provides fast-acting and efficacious relief of ETTH. Both IBU_Na_ and standard IBU were significantly better than placebo for all efficacy parameters studied, and there were no AEs in any treatment group. Median FPR was nearly 7 minutes faster and median MPR was approximately 8 minutes faster with IBU_Na_ than standard IBU tablets. Although these differences were not statistically significant using the prespecified statistical analyses, they were statistically significant in post hoc analyses using a methodology that assigns higher weights to earlier events. In addition, significantly more subjects achieved FPR within 30 minutes with IBU_Na_ versus standard IBU. Thus, this formulation of IBU_Na_ is as safe and effective as standard IBU and provides fast-acting relief of ETTH.

## Abbreviations

AEs: Adverse events
ANOVA: Analysis of variance
CI: Confidence interval
CMH: Cochran-Mantel-Haenszel
ETTH: Episodic tension-type headache
FPR: First perceptible pain relief
HR: Hazard ratio
IBU: Ibuprofen
IBU_Mot_: Motrin®
IBU_Na_: Ibuprofen sodium dihydrate
ITT: Intent-to-treat
MedDRA: Medical Dictionary for Regulatory Activities
MPR: Meaningful pain relief
NA: Not applicable
NSAID: Nonsteroidal anti-inflammatory drug
OTC: Over-the-counter
PID: Pain Intensity Difference
PRID: Sum of Pain Relief Rating and Pain Intensity Difference scores
PRR: Pain Relief Rating
PSR: Pain Severity Rating
SD: Standard deviation
SE: Standard error
SPID: Sum of Pain Intensity Difference scores
SPRID: Mean time-weighted sum of Pain Relief Rating and Pain Intensity Difference scores
TOTPAR: Time-weighted sum of Pain Relief Rating scores
VAS: Visual analog scale

## References

[1] Headache Classification Subcommittee of the International Headache Society (2004). The International Classification of Headache Disorders, 2nd ed. Cephalalgia.

[2] Advil [package insert]. Madison, NJ: Pfizer Consumer Healthcare; 2012.

[3] Schachtel BP, Furey SA, Thoden WR (1996). Nonprescription ibuprofen and acetaminophen in the treatment of tension-type headache. J Clin Pharmacol.

[4] Cooper SA, Needle SE, Kruger GO (1977). Comparative analgesic potency of aspirin and ibuprofen. J Oral Surg.

[5] Cooper SA, Schachtel BP, Goldman E, Gelb S, Cohn P (1989). Ibuprofen and acetaminophen in the relief of acute pain: a randomized, double-blind, placebo-controlled study. J Clin Pharmacol.

[6] Schachtel BP, Fillingim JM, Thoden WR, Lane AC, Baybutt RI (1988). Sore throat pain in the evaluation of mild analgesics. Clin Pharmacol Ther.

[7] Schachtel BP, Thoden WR, Baybutt RI (1989). Ibuprofen and acetaminophen in the relief of postpartum episiotomy pain. J Clin Pharmacol.

[8] Laska EM, Sunshine A, Marrero I, Olson N, Siegel C, McCormick N (1986). The correlation between blood levels of ibuprofen and clinical analgesic response. Clin Pharmacol Ther.

[9] Black P, Max MB, Desjardins P, Norwood T, Ardia A, Pallotta T (2002). A randomized, double-blind, placebo-controlled comparison of the analgesic efficacy, onset of action, and tolerability of ibuprofen arginate and ibuprofen in postoperative dental pain. Clin Ther.

[10] Schettler T, Paris S, Pellett M, Kidner S, Wilkinson D (2001). Comparative pharmacokinetics of two fast-dissolving oral ibuprofen formulations and a regular-release ibuprofen tablet in healthy volunteers. Clin Drug Investig.

[11] Desjardins P, Black P, Papageorge M, Norwood T, Shen DD, Norris L (2002). Ibuprofen arginate provides effective relief from postoperative dental pain with a more rapid onset of action than ibuprofen. Eur J Clin Pharmacol.

[12] Sorgel F, Fuhr U, Minic M, Siegmund M, Maares J, Jetter A (2005). Pharmacokinetics of ibuprofen sodium dihydrate and gastrointestinal tolerability of short-term treatment with a novel, rapidly absorbed formulation. Int J Clin Pharmacol Ther.

[13] Mehlisch DR, Ardia A, Pallotta T (2002). A controlled comparative study of ibuprofen arginate versus conventional ibuprofen in the treatment of postoperative dental pain. J Clin Pharmacol.

[14] Schleier P, Prochnau A, Schmidt-Westhausen AM, Peters H, Becker J, Latz T (2007). Ibuprofen sodium dihydrate, an ibuprofen formulation with improved absorption characteristics, provides faster and greater pain relief than ibuprofen acid. Int J Clin Pharmacol Ther.

[15] Norholt SE, Hallmer F, Hartlev J, Pallesen L, Blomlof J, Hansen EJ (2011). Analgesic efficacy with rapidly absorbed ibuprofen sodium dihydrate in postsurgical dental pain: results from the randomized QUIKK trial. Int J Clin Pharmacol Ther.

[16] Kellstein D, Legg TJ, Leyva R (2013). A novel formulation of ibuprofen sodium is absorbed faster than standard ibuprofen tablets [abstract 388]. J Pain.

[17] Brain P, Leyva R, Doyle G, Kellstein D. Onset of analgesia and efficacy of ibuprofen sodium in postsurgical dental pain: a randomized, placebo-controlled study versus standard ibuprofen. Clin J Pain. 2014 Aug 27. [Epub ahead of print]. 10.1097/AJP.0000000000000142 PMC4388398 25119511

[18] Guidance for industry analgesic indications: developing drug and biological products. U.S. Department of Health and Human Service, Food and Drug Administration, Center for Drug Evaluation and Research. http://www.fda.gov/downloads/drugs/guidancecomplianceregulatoryinformation/guidances/ucm384691.pdf#page=1&zoom=auto,-190,792. Accessed Aug 20, 2014.

[19] Packman B, Packman E, Doyle G, Cooper S, Ashraf E, Koronkiewicz K (2000). Solubilized ibuprofen: evaluation of onset, relief, and safety of a novel formulation in the treatment of episodic tension-type headache. Headache.

[20] Diamond S, Balm TK, Freitag FG (2000). Ibuprofen plus caffeine in the treatment of tension-type headache. Clin Pharmacol Ther.

[21] Moller PL, Sindet-Pedersen S, Petersen CT, Juhl GI, Dillenschneider A, Skoglund LA (2005). Onset of acetaminophen analgesia: comparison of oral and intravenous routes after third molar surgery. Br J Anaesth.

[22] Cooper SA, Voelker M (2012). Evaluation of onset of pain relief from micronized aspirin in a dental pain model. Inflammopharmacology.

[23] Wininger SJ, Miller H, Minkowitz HS, Royal MA, Ang RY, Breitmeyer JB (2010). A randomized, double-blind, placebo-controlled, multicenter, repeat-dose study of two intravenous acetaminophen dosing regimens for the treatment of pain after abdominal laparoscopic surgery. Clin Ther.

[24] Averbuch M, Katzper M (2003). Severity of baseline pain and degree of analgesia in the third molar post-extraction dental pain model. Anesth Analg.

[25] Rasmussen BK, Jensen R, Olesen J (1992). Impact of headache on sickness absence and utilisation of medical services: a Danish population study. J Epidemiol Community Health.

